# Morning fatigue and structured exercise interact to affect non-exercise physical activity of fit and healthy older adults

**DOI:** 10.1186/s12877-021-02131-y

**Published:** 2021-03-12

**Authors:** Tomas Vetrovsky, Dan Omcirk, Jan Malecek, Petr Stastny, Michal Steffl, James J. Tufano

**Affiliations:** 1 Department of Physiology and Biochemistry, Faculty of Physical Education and Sport, Charles University, Prague, Jose Martiho 269/31, 162 52 Prague, Czech Republic; 2 Department of Sport Games, Faculty of Physical Education and Sport, Charles University, Prague, Czech Republic

**Keywords:** Within-subject analysis, Accelerometer, Ecological momentary assessment, Intensity gradient, Physical activity compensation

## Abstract

**Background:**

Exercise training is crucial for maintaining physical and mental health in aging populations. However, as people participate in structured exercise training, they tend to behaviorally compensate by decreasing their non-exercise physical activity, thus potentially blunting the benefits of the training program. Furthermore, physical activity of older adults is substantially influenced by physical feelings such as fatigue. Nevertheless, how older people react to day-to-day fluctuations of fatigue and whether fatigue plays a role in non-exercise physical activity compensation is not known. Thus, the purpose of this study was twofold: (1) To explore whether the volume and intensity of habitual physical activity in older adults were affected by morning fatigue. (2) To investigate the effect of attending power and resistance exercise sessions on the levels of non-exercise physical activity later that day and the following day.

**Methods:**

Twenty-eight older adults wore an accelerometer during a 4-week low-volume, low-intensity resistance and power training program with three exercise sessions per week and for 3 weeks preceding and 1 week following the program. During the same period, the participants were prompted every morning, using text messages, to rate their momentary fatigue on a scale from 0 to 10.

**Results:**

Greater morning fatigue was associated with lower volume (*p* = 0.002) and intensity (*p* = 0.017) of daily physical activity. Specifically, one point greater on the fatigue scale was associated with 3.2 min (SE 1.0) less moderate-to-vigorous physical activity. Furthermore, attending an exercise session was associated with less moderate-to-vigorous physical activity later that day by 3.7 min (SE 1.9, *p* = 0.049) compared to days without an exercise session. During the next day, the volume of physical activity was greater, but only in participants with a body mass index up to 23 (*p* = 0.008).

**Conclusions:**

Following low-volume exercise sessions, fit and healthy older adults decreased their non-exercise physical activity later that day, but this compensation did not carry over into the next day. As momentary morning fatigue negatively affects daily physical activity, we suggest that the state level of fatigue should be monitored during intensive exercise programs, especially in less fit older adults with increased fatigability.

## Background

Physical activity (PA) and exercise are crucial for maintaining physical and mental health in aging populations [1–3], and various exercise interventions have been shown to increase physical fitness, function, and well-being of older adults [4–6]. However, as early as the 1990s, researchers noticed that as people participate in structured exercise training, they tend to behaviorally compensate by decreasing their non-exercise PA (NEPA) [7, 8]. Since then, many studies have explored this phenomenon, resulting in conflicting data. For example, a recent review found that NEPA compensation was observed in only four out of twelve exercise studies that included a variety of training programs [9]. However, most of the studies where NEPA compensation was not present were not designed to detect short-term within-subject changes in NEPA, which may occur on a day-to-day basis and could accumulate into clinically meaningful changes. Furthermore, it is not clear whether aerobic and resistance training have the same effect on NEPA. Interestingly, one small study suggested that the compensatory reduction in NEPA in response to resistance exercise is much smaller than during aerobic exercise [10]. Nevertheless, there are too few studies exploring the effect of resistance training on NEPA [7, 11] to draw any meaningful conclusions about the role of the exercise mode. Finally, yet another systematic review with meta-analysis indicated that, on average, NEPA did not change in response to exercise training programs [12]. Having said that, the authors also reported that longer session duration, shorter intervention length, and older age may have played a role in decreasing NEPA during exercise training programs [12]. In fact, earlier studies [13, 14] also indicated that older adults may be more susceptible to the negative effect of exercise training on NEPA.

The functional status and PA of older adults are substantially influenced by physical feelings such as fatigue [15, 16], and fatigue might therefore play a substantial role in NEPA compensation following exercise in older populations. Admittedly, older adults typically experience higher levels of fatigue [17–19], and several cross-sectional studies have demonstrated that older adults who reported feeling more fatigued took fewer steps per day and had lower minutes of moderate-to-vigorous PA (MVPA) [20, 21]. Nevertheless, the relationship between fatigue and PA in older adults is likely bi-directional [21]. Additionally, in longitudinal analyses, those who reported feeling less fatigued over time had greater increases in PA [21]. Although these studies indicate a tight connection between fatigue and PA, they mostly explored between-subject associations where fatigue was measured as a trait characteristic (i.e., their usual, average level of fatigue). However, studies analyzing within-subject associations between momentary state fatigue (i.e., the amount of fatigue at a specific moment in time) and PA are needed to elucidate how older people react to day-to-day fluctuations of fatigue and to determine whether fatigue plays a role in NEPA compensation following exercise training [22, 23]. Additionally, analyzing the day-to-day fluctuations of state fatigue and PA within subjects is especially suitable for detecting the short-term impact of exercise on NEPA compensation within a given day, even if the compensation does not translate into the next day.

Thus, the objective of our study was twofold: (1) to explore whether the volume and intensity of daily habitual PA in older adults were affected by momentary morning fatigue (objective #1), and (2) to investigate the effect of attending power and resistance training sessions on the levels of NEPA later that day (objective #2a) and the following day (objective #2b).

## Methods

### Design and participants

This prospective observational study was conducted alongside a training study that included a 4-week training program consisting of three structured power and resistance training sessions per week [24]. Habitual PA and momentary morning fatigue were assessed daily during the training program, as well as for 3 weeks preceding and 1 week following the program.

Participants were recruited from a local senior club and were eligible if they: (1) were over 60 years old, (2) provided written informed consent, (3) provided signed consent from their general practitioner that they could participate in the training program, and (4) were willing to use their mobile phone to receive and answer text messages as part of the study. Participants with co-morbid conditions that would affect adherence to the training program or study procedures (e.g., major depression or other significant psychiatric disorders, dementia or cognitive impairment, significant hearing or a visual impairment, or inability to walk from any reason) were excluded from the study.

### Procedures

At baseline, participants’ anthropometric measures (body mass, height) were taken, and their functional status was assessed by a six-minute walk test.

Following the baseline assessment, participants were fitted with an Actigraph wGT3X-BT accelerometer (ActiGraph, Pensacola, FL, USA) and were requested to wear it for three consecutive weeks (pre-training period). The accelerometer was attached to the right hip using an elastic band provided by the manufacturer. Participants were instructed to remove it only when sleeping at night and for water-based activities.

During the same 3-week pre-training period, every morning at 8:45 am (9:45 am on weekends), participants received a text message (SMS) asking them to rate their momentary level of fatigue using a scale from 0 to 10 where 0 = no fatigue and 10 = as fatigued as you can imagine. This single-item global fatigue severity scale was taken from the Brief Fatigue Inventory [25] and has been validated in older adults [26]. It is commonly used in ecological momentary assessment studies [27–29], including those in older adults [30, 31], as a measure of the state level of fatigue. Participants were requested to answer the text message immediately, or as soon as they safely could (e.g., they were explicitly instructed not to answer the text message while driving). Only answers received before noon were considered valid and included in the analysis.

After returning the accelerometer at the end of the pre-training period, participants were left without an accelerometer and text messages for 1 week, before starting the training period. This week-long interruption in accelerometer wear was intended for charging the devices and checking their correct function, as well as for enabling the participant to rest from adhering to the protocol.

The 4-week training period included training sessions on Mondays, Wednesdays, and Fridays, always between 8:00 am and 2:00 pm. The individual training sessions were supervised by fitness trainers, lasted approximately 20 min, and consisted of a warm-up, lower body power training, and upper body resistance band strengthening. The lower body power training included maximum effort countermovement jumps, either with the participant’s bodyweight or assisted [32, 33], with the number of jumps per session starting at five jumps and progressing up to 24 jumps. The upper body resistance band strengthening consisted of three sets of 10 repetitions of overhead press, lat pull down, chest press, single-arm row, and seated rotation. Describing the training program in detail is outside of the scope of this paper, but the average rating of perceived exertion during the training sessions was 3 out of 10. Therefore, the training sessions were not overly exhausting but included novel exercise stimuli that resulted in some degree of acute fatigue and performance adaptations [24].

During the training period, participants wore the accelerometer and received the daily morning text messages on their momentary level of fatigue in the same way as they did during the pre-training period.

Following the training period, participants were left without an accelerometer and text messages for 1 week, before starting the post-training period, during which they once again wore the accelerometer and received the daily text messages on their level of fatigue as in the pre-training period.

### Physical activity measures

To explore whether daily PA was affected by morning fatigue (objective #1) and by participating in a training session the previous day (objective #2b), we analyzed 12-h time windows between 9:00 am and 9:00 pm which reflected the sleep pattern of most participants which we ascertained during the interviews at baseline. Accordingly, the text messages on morning fatigue were sent out right before the start of this time window (with the exception of weekends when we were careful not to wake up the participants and sent it 1 hour later).

To analyze the effect of attending the training session on PA levels later that day (objective #2a), we chose the 6-h time window between 3:00 pm and 9:00 pm (termed as “the second half of the day” throughout this paper) because all training sessions were finished by 2:00 pm and we allowed an additional hour for commuting back home to ensure that potential active commuting (e.g., walking or cycling) related to the training session did not influence PA levels entering the analysis.

For primary analysis of the effect of training sessions and fatigue on PA, we used average acceleration (ACC) as a measure of activity volume, and we used intensity gradient (IG) as a measure of activity intensity [34]. Together, these novel metrics provide a complementary description of a person’s activity profile, and unlike more traditional measures of PA, such as minutes of MVPA, they are not cut-point dependent and thus are more sensitive to subtle changes in PA [35]. Specifically, even substantial increases in measured acceleration might not have an impact on the number of MVPA minutes if those minutes do not cross the cut-point for moderate PA. In addition, the cut-points are protocol- and population-specific, and using different cut-points can lead to wildly discrepant results [36]. Despite the drawbacks of using the minutes of MVPA, we still preferred to report them in addition to ACC and IG, as they are easily understood and facilitate comparison with other studies. However, they should be interpreted with caution.

Using the ActiLife 6 (v6.13.3) software provided by the manufacturer (ActiGraph, Pensacola, FL, USA), the data were downloaded from accelerometers and exported as raw acceleration (sampling frequency 30 Hz) into csv files. The csv files were then processed in the GGIR R-package according to standard procedures, including detection of non-wear, and calculation of the average magnitude of dynamic acceleration corrected for gravity (Euclidean Norm minus 1 g) over 5-s epochs [37]. However, unlike the standard procedure that imputes missing data (e.g., non-wear) by the average at similar time points on different days of the week, we did not perform the imputation *(do.imp = FALSE)*, as it would have affected the day-to-day variability.

For each of the metrics (ACC, IG, MVPA), we calculated their values for time windows between 9 am and 9 pm *(qwindow = c* [9, 21]*)*, and between 3 pm and 9 pm *(qwindow = c* [15, 21]*)*. To calculate minutes of MVPA, we used the cut point of 70 mg *(mvpathreshold = 70)* [38]. As ACC and IG metrics are sensitive to non-wear time, only days with full 12 and 6 valid hours, respectively, were included in the analysis of ACC and IG. For MVPA, days with at least 10 and 5 valid hours, respectively, were included in the analysis, which is in line with recommended best practices [39]. Of note, applying the ACC and IG metrics to a 6 and 12-h time window means that their values reported in the Results section of this paper are not directly comparable with the results of other studies where both metrics usually describe PA of the whole 24-h day.

### Statistical analysis

To examine the effects of morning fatigue (objective #1) and participating in a training session (objective #2) on PA, we conducted a series of linear mixed-effects models using the lme4 (version 1.1–20) and lmerTest (version 3.1–0) packages in R, version 3.5.2 (The R Foundation for Statistical Computing, Vienna, Austria).

For each of the PA metrics (ACC, IG, MVPA), the full model included the explanatory variable of interest (morning fatigue for objective #1 and participation in a training session for objective #2) in interactions with potential covariates (gender, age, BMI, day of the week, and morning fatigue (only for objective #2)) as fixed effects. Participant and day of measurement nested within the period of measurement (pre-training, training, post-training) entered the model as random effects.

The covariates and their interactions that did not demonstrate significant associations (*p* < 0.05) with the PA metric were eliminated from the full model one by one, and only those with significant associations were included in the presented final model for that specific metric. Thus, each of the final models included a different set of covariates. The residual plots of final models were visually inspected to verify the assumptions of homoscedasticity and normality. When the final models included an interaction between the explanatory variable of interest and any of the covariates, we performed post-hoc general linear hypotheses testing of the final model using the multcomp (version 1.4–8) package in R to determine the effect of selected values of the covariate.

In models examining the effect of morning fatigue on PA (objective #1), only data from the pre-training and post-training period were used to eliminate the effect of training sessions on morning fatigue. In models investigating the effect of participation in a training session on PA in the next day (objective #2b), only data from days without training session were used to eliminate the effect of accelerometer removal during the training session and the effect of commuting to and from the training session on PA in that day. Finally, the level of morning fatigue was modeled as a function of participation in a training session in the previous day. Only data from days without a training session were used in this model to make sure that an early morning session (that could start as early as 8:00 am) did not impact the reported level of fatigue.

## Results

Twenty-eight participants were recruited for the study (their characteristics are depicted in Table 1). During the training period, two participants got sick after attending the first three and six training sessions, respectively, and withdrew from the study. Regardless, their data until the withdrawal were included in the analysis. The remaining 26 participants attended between 7 (58%) and 12 (100%) training sessions (median 11 (92%)). Due to the technical failure of accelerometers, no PA data were available for one participant from the pre-training period, four participants from the training period, and two participants from the post-training period. Furthermore, one participant could not answer the text messages about morning fatigue, but that participant’s PA data were included in the analysis. The average response rate of the remaining 27 participants to the text message about morning fatigue was 87 ± 17%.

**Table 1:** Participant characteristics

	*N* = 28
Age, years	68 ± 7
Female	21 (75%)
Male	7 (25%)
Weight, kg	71.8 ± 13.7
Body mass index, kg/m^2^	25.8 ± 3.6
Six-minute walk distance, m	647 ± 82
Daily minutes of moderate-to-vigorous physical activity	54 ± 38
Mean fatigue score (scale from 0 to 10)	1.90 ± 1.68

Data are presented as mean ± SD or number (percentage)

Altogether, 1618 participant-days were included in the analysis; of those, 269 were days with a training session. PA data were available for 1479 days; of those, at least 10 h (for the analysis of MVPA) and 12 h (for the analysis of ACC and IG) of valid data were available for 1311 days (89 excluded) and 1107 days (372 excluded), respectively, and at least 5 h (for the analysis of MVPA) and 6 h (for the analysis of ACC and IG) in the second half of the day were available for 1298 days (181 excluded) and 1219 days (260 excluded), respectively. Valid data on morning fatigue were available for 1390 days. The number of days entering the individual models is reported in the headings of Tables 2, 3 and 4.

**Table 2:** Daily physical activity as a function of morning fatigue

Fixed effects	ACC (*N* = 525)		IG (*N* = 525)		MVPA (*N* = 631)	
	β (SE)	*p*-value	β (SE)	*p*-value	β (SE)	*p*-value
(Intercept)	79.01 (15.17)		0.55 (0.49)		233.8 (31.5)	
Fatigue	21.24 (6.76)	0.002	0.67 (0.28)	0.017	−3.2 (1.0)	0.002
Age	−0.74 (0.19)	< 0.001	− 0.03 (0.01)	< 0.001	−2.6 (0.5)	< 0.001
BMI	−0.09 (0.37)	0.818	−0.03 (0.01)	0.005	NA	NA
Fatigue*Age	−0.24 (0.08)	0.004	−0.01 (0.00)	0.013	NA	NA
Fatigue*BMI	−0.23 (0.09)	0.012	NA	NA	NA	NA

The asterisk (*) signifies an interaction between variables. N is the number of days entering the individual models. *ACC* average acceleration in milligravitational units (mg) represents the volume of physical activity, *IG* intensity gradient represents the intensity of physical activity, *MVPA* minutes of moderate to vigorous physical activity, *BMI* body mass index, *NA* not assessed, i.e., the covariate was not included in the final model

**Table 3:** Physical activity in the second half of the day as a function of participation in a training session earlier that day

Fixed effects	ACC (*N* = 1219)		IG (*N* = 1219)		MVPA (*N* = 1298)	
	β (SE)	*p*-value	β (SE)	*p*-value	β (SE)	*p*-value
(Intercept)	63.38 (13.98)		0.10 (0.46)		90.9 (17.7)	
Session	NA	NA	−0.14 (0.05)	0.009	−3.7 (1.9)	0.049
Age	−0.61 (0.20)	0.006	−0.04 (0.01)	< 0.001	−1.0 (0.3)	< 0.001
Day of the week	NA	NA		0.015		0.016

*N* is the number of days entering the individual models. *ACC* average acceleration in milligravitational units (mg) represents the volume of physical activity, *IG* intensity gradient represents the intensity of physical activity, *MVPA* minutes of moderate to vigorous physical activity, *NA* not assessed, i.e., the covariate was not included in the final model

**Table 4:** Daily physical activity as a function of participating in a training session in the previous day

	ACC (*N* = 806)		IG (*N* = 910)		MVPA (*N* = 960)	
Fixed effects	β (SE)	*p*-value	β (SE)	*p*-value	β (SE)	*p*-value
(Intercept)	89.00 (15.71)		0.53 (0.47)		263.0 (39.3)	
Session	11.18 (4.57)	0.015	NA	NA	48.1 (17.5)	0.006
Fatigue	−0.75 (0.22)	< 0.001	NA	NA	−3.0 (0.8)	< 0.001
BMI	−0.42 (0.36)	0.093	−0.03 (0.01)	0.006	−1.2 (0.9)	0.029
Age	−0.76 (0.20)	< 0.001	−0.03 (0.01)	< 0.001	−2.5 (0.5)	< 0.001
Session*BMI	−0.40 (0.18)	0.024	NA	NA	−1.7 (0.7)	0.013

The asterisk (*) signifies an interaction between variables. *N* is the number of days entering the individual models, *ACC* average acceleration in milligravitational units (mg) represents the volume of physical activity, *IG* intensity gradient represents the intensity of physical activity, *MVPA* minutes of moderate to vigorous physical activity, *BMI* body mass index, *NA* not assessed, i.e., the covariate was not included in the final model

### Objective #1

**Fig. 1:**
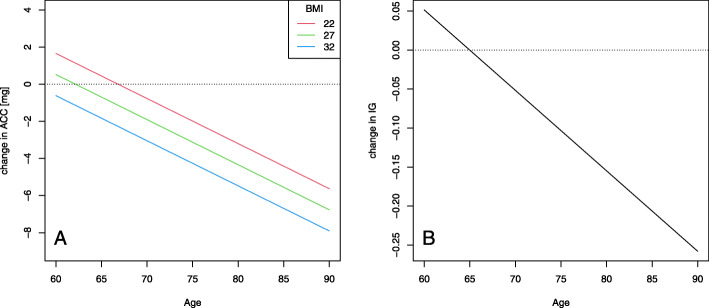
Greater morning fatigue is associated with less physical activity later that day. Legend: The effect of greater morning fatigue by 1 point (on a scale from 0 to 10) on physical activity as a function of age and BMI. **a** The effect on ACC is moderated both by age and body mass index: the lines represent BMI 22, 27, and 32, respectively; (**b**) The effect on IG is moderated by age. ACC: average acceleration in milligravitational units (mg) represents the volume of physical activity. IG: intensity gradient represents the intensity of physical activity. BMI: body mass index

Greater morning fatigue was associated with lower ACC and IG (Table 2). The effect of fatigue was moderated by age (for both ACC and IG) and BMI (only for ACC): with greater age and BMI, the association between greater fatigue and less PA was more pronounced (Fig. 1). Greater morning fatigue was also associated with less time spent in MVPA, though this effect was not moderated either by age or by BMI. Specifically, one point higher on the fatigue scale was associated with 3.2 min (SE 1.0) less MVPA (Table 2).

### Objective #2a

Participating in a training session in the first half of the day was associated with lower IG (but not ACC) in the second half of the day (Table 3). Furthermore, participating in a training session in the first half of the day was also associated with lower MVPA by 3.7 min (SE 1.9) in the second half of the day compared to days without an exercise session (Table 3).

### Objective #2b

**Fig. 2:**
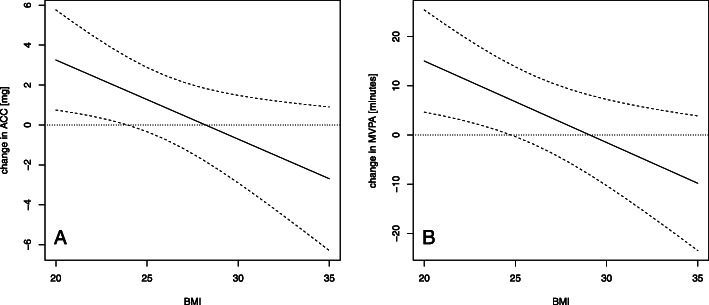
Participation in a training session and physical activity during the next day. Legend: The effect of participation in a training session on physical activity during the next day as a function of body mass index. **a** The effect on ACC; **b** The effect on minutes of MVPA. The dashed lines represent two standard errors. ACC: average acceleration in milligravitational units (mg) represents the volume of physical activity. MVPA: minutes of moderate to vigorous physical activity. BMI: body mass index

The association between participating in a training session and ACC and MVPA during the day following the training session was moderated by BMI (Table 4). Specifically, for lower BMI (up to 28 for ACC and 29 for MVPA), participating in a training session was associated with greater ACC and MVPA during the next day, while for greater BMI, participating in a training session was associated with less ACC and MVPA during the next day (Fig. 2). However, as demonstrated in the post-hoc analysis, the association was statistically significant only for low BMI (up to 23 for ACC and 24 for MVPA). For example, for a model participant with a BMI of 22, participating in a training session was associated with 11.7 min (SE 4.4, *p* = 0.008) more MVPA during the next day compared to days without an exercise session according to the model. There was no association between participating in a training session and IG during the next day (Table 4).

Furthermore, when the level of morning fatigue was modeled as a function of participation in a training session during the previous day, the model demonstrated that participating in a training session was associated with a lower fatigue by 0.37 points (SE 0.13, *p* = 0.004) on the fatigue scale in the morning of the next day.

## Discussion

### Fatigue and PA (objective #1)

This study demonstrated a within-subject association between greater morning fatigue and lower volume and intensity of daily PA in healthy adults over 60 years of age (objective #1). Specifically, an increase of one point on the fatigue scale (from 0 to 10) was associated with 3.2 min less MVPA per day, which translates into 22 min less MVPA per week. This can be considered as a clinically significant effect, given that 22 min equates to roughly 15% of the recommended weekly minimum goal of 150 min of MVPA [1].

Our findings are consistent with the results of two studies in middle-aged adults [29] and patients with osteoarthrosis [40] which found that fatigue was a strong predictor of reduced PA in the subsequent 15 min and 4 hours after training, respectively. In agreement with these studies, our finding that greater morning fatigue was associated with lower levels of PA later that day suggests a possible causal relationship between fatigue and PA. This is a novel finding that extends on the results of previous cross-sectional and longitudinal studies in older adults that explored these associations between-, rather than within-, subjects, and therefore could not unveil the temporal sequence of fatigue and PA as was possible in the present study [20, 21, 41]. Nevertheless, the relationship between fatigue and PA in older adults is likely bi-directional, because other studies indicated that greater PA throughout a day resulted in greater fatigue in the evening [42].

Furthermore, our data revealed that the association between greater morning fatigue and lower daily PA was moderated by age and BMI; that is, with greater age and BMI, the association between greater fatigue and less PA was more profound. This finding is particularly important, given that as people get older, their PA levels decrease [43] and their fatigue levels increase [19]. Thus, our finding suggests that the decrease of PA at older ages could be partially caused by the higher sensitivity of older people to fatigue. However, our study was not designed to answer this question, and future longitudinal studies are needed for a definitive answer. The moderating effect of BMI is also of concern because it contributes to the vicious cycle of obesity and physical inactivity, and it is likely responsible for the compensation of habitual PA in some weight-loss programs and the resulting suboptimal weight loss during that time [44, 45]. While our sample size was too small to make conclusive statements about the moderating effect of age and BMI, this finding opens the door for future research on the topic.

### Structured training and NEPA (objective #2)

Our study found that healthy and relatively fit older adults participating in resistance and power training sessions in the first half of the day compensated by decreasing the intensity, but not the volume, of NEPA in the second half of the same day (objective #2a). However, this NEPA compensation did not carry over into the next day. Moreover, the participants with lower BMI scores actually had a greater PA volume during the next day, which could be partially explained by less morning fatigue (objective #2b). Collectively, these findings demonstrate that with a relatively small, yet progressive increase in training volume, the potential compensation was limited to the training day and did not impact the following days. This might help to explain why so many previous studies failed to detect any compensation in NEPA despite exposing the participants to a much greater training volume [9, 12]. As those studies compared PA before and after the entire training period rather than measuring its day-to-day fluctuations, they could have easily missed the same-day effect of compensation. Indeed, it seems that a within-subject design analyzing NEPA on individual days is better suited to detect short-term behavioral compensation than studies comparing longer periods of time before and after a training program. Thus, our study is a good illustration of how a within-subject repeated-measures design can shed light on phenomena that include large day-to-day variations, such as the relationship between exercise training and NEPA.

Unfortunately, the design of our study does not help in explaining the reasons for the compensation in the second half of the day. We do not know if it was due to training-induced fatigue, psychological causes (“I already exercised, so now, I can afford to relax.”), organizational reasons (the need to catch up with the activities they would have otherwise done in time of the training session), or a combination of factors. Nevertheless, we can speculate that fatigue played some role here, as the participants compensated in the intensity but not volume of PA after training, which is in line with findings of other studies noting that with increased fatigue, older adults decrease their walking speed [15, 46]. However, further studies, including repeated assessments of fatigue throughout the day, are needed to elucidate the role of fatigue in the decrease of NEPA in the second half of the day.

Our finding that the participants with low BMI had greater levels of NEPA in the next day following the training session is novel and somewhat surprising. Of note, the greater levels of NEPA were associated with less morning fatigue. Thus, less fatigue could partially explain the greater levels of NEPA, at least in very old adults whose NEPA is susceptible to the state levels of morning fatigue as illustrated in Fig. 1. For example, in a model 80-year-old participant with a BMI of 22, a 0.37 points lower fatigue score was associated with greater ACC by 1.12 mg (based on β coefficients in Table 2) which is about a half of the difference in NEPA for this model participant (based on β coefficients in Table 4). This calculation illustrates that in very old adults with low BMI, less morning fatigue might be partially responsible for the greater NEPA in the day following the training session.

The lower morning fatigue following the training day, which was observed in all participants regardless of their age or BMI, is in line with another study where an 18-month exercise intervention reduced fatigue in overweight and obese adults aged 55 years and older [21]; however, there is no clear explanation for these observations. One possibility is that the lower NEPA in the second half of the training day was of such an extent that it not only eliminated the exercise-induced fatigue, but also contributed to less fatigue in the next morning. Another explanation could be that participating in the training led to a better and more restorative sleep, and the participants woke up less fatigued the next day. However, these ideas were not assessed in the present study, and further studies assessing sleep, for example by using a wrist-worn accelerometer over 24-h a day, would help to confirm or reject this hypothesis.

The fact that the compensation occurred in the second half of the day following the training, but not the next day, raises an interesting question of what would happen if the training was scheduled during the second half of the day. On one hand, it might eliminate the compensation observed in our study (provided the night’s sleep would be sufficiently restorative in relation to the training volume). On the other hand, attending the training session in the second half of the day when older adults usually experience increased levels of fatigue might lead to suboptimal performance during the training and hamper its beneficial effects. Therefore, a study comparing morning and evening training sessions in relation to NEPA would be interesting, as it could provide information regarding the optimal timing of resistance and power training sessions in older adults.

The training program employed in our study consisted of lower body power training (i.e., maximum effort countermovement jumps) and upper body resistance training using elastic bands. Exercise modality (e.g., aerobic vs. resistance training) is a factor that could influence changes in NEPA during exercise training, but its role is not clear. The effect of resistance training on NEPA has been explored by a handful of studies, but their results are inconclusive [7, 11, 14, 47]. Furthermore, two studies directly compared aerobic and resistance training with respect to NEPA. One of them, a larger between-subject study, evaluated NEPA before and after an 8-month intervention in 82 middle-aged adults participating in aerobic, resistance, or combined training programs, but it did not find any differences between the exercise modalities [48]. However, the other, smaller within-subject, study suggested that resistance training might actually increase, rather than decrease, NEPA [10]. Specifically, using a cross-over design, the study compared 16-week aerobic and resistance training programs in nine young men and found that non-exercise MVPA decreased on training days during the aerobic program but not during the resistance program [10]. Furthermore, they found that on non-training days during the resistance program (but not aerobic program), MVPA actually increased, which is in line with our observation of greater PA in the day following the training session in participants with a lower BMI. Thus, our study supports the view that an appropriate dose of resistance training could have a positive rather than negative impact on NEPA, at least in healthy older adults with lower BMI scores. However, whether this is true and what is the appropriate dose for various populations remains to be explored in future studies.

### Practical considerations and limitations

The finding that the NEPA compensation did not carry over into the next day might sound comforting for practitioners designing training programs for older adults. However, when considering generalizability of our results, several limitations of the study need to be taken into account. First, the study population was highly active (54 min of MVPA per day), had high level of physical fitness for their age (647 m walked during the six-minute walk test), and reported very low levels of momentary morning fatigue (1.9 on average on a scale from 0 to 10). As the negative impact of fatigue is greater with greater age and BMI (Fig. 1), we might speculate that more sedentary and unfit older adults would demonstrate an even more detrimental impact of fatigue on their PA. As such, the findings of this study cannot be generalized to sedentary and less fit populations with greater levels of fatigue, and practitioners need to be cautious about the possible negative effects of exercise training on NEPA in these populations. Nevertheless, healthy active older adults are not always as well-represented as their sedentary or less-fit counterparts. Thus, although our results may not be directly relevant for the majority of sedentary older adults, these data on healthy older adults should not be undervalued. Second, our study did not assess day-to-day variability of sleep and depressive symptoms that are both important factors strongly related to fatigue. Thus, we cannot exclude that the observed effects of fatigue were not confounded by poor sleep or worsening of depressive symptoms. Third, as the assessment of momentary morning fatigue was not individualized but conducted at a fixed time of the day (at 8:45 am on weekdays and at 9:45 am on weekends), it might be possible that participants occasionally performed some high-effort activity between their awakening and fatigue assessment (e.g., morning jog) which could affect their assessment of fatigue. While we did not ask the participants specifically about their morning activities, we visually inspected their accelerometer data to make sure that they mostly just performed normal activities of daily living without any high-intensity bursts of physical activity. Fourth, our study did not assess participants’ perceived fatigability which is a more sensitive measure of fatigue than self-reported fatigue as it is not subject to self-pacing bias [40, 41]. Thus, we cannot exclude that the observed moderating effects of age and BMI were not confounded by differences in fatigability. Fifth, even though the low-volume (20 min per session), low-intensity (rating of perceived exertion of 3 out of 10) training program of the present study resulted in significant performance adaptations [24], it is possible that the program was not stressful enough to largely affect fatigue and PA in our active and fit population. Therefore, more intense exercise could have a greater effect, and when applied to less fit older adults or those with chronic conditions, it might result in such decreases in NEPA that would eventually carry over into the following days. Finally, as discussed in the previous paragraph, aerobic training might have a more negative effect on NEPA than the power and resistance training that we employed in our study. Thus, until future studies explore the day-to-day variations in NEPA following aerobic training, practitioners need to be careful when applying the results of our study to other training modalities.

## Conclusions

With a relatively low, yet progressively increasing, volume of power and resistance training, fit and healthy older adults compensated through decreasing NEPA later that day, but this NEPA compensation did not carry over into the next day. However, as morning fatigue negatively affects daily PA, interventions with inappropriately high training volume leading to a substantial increase in state fatigue might result in a more prolonged decrease of NEPA, thus blunting the benefits of the training program. Consequently, we suggest that the state level of fatigue should be monitored during intensive interventions, especially in less fit older adults and those with chronic conditions associated with increased fatigability.

## Data Availability

The datasets used and/or analyzed during the current study are available from the corresponding author on reasonable request.

## Abbreviations

PA: Physical activity
NEPA: Non-exercise physical activity
MVPA: Moderate-to-vigorous physical activity
ACC: Average acceleration
IG: Intensity gradient
BMI: Body mass index
